# Could Interscalene Block Possibly be Protective Against Cerebral Ischemia During Shoulder Surgery in a Beach Chair Position?

**DOI:** 10.7759/cureus.16773

**Published:** 2021-07-31

**Authors:** Sami Kaan Coşarcan, Yavuz Gurkan, Alper Tunga Doğan, Özgür Koyuncu, Ömür Erçelen

**Affiliations:** 1 Anesthesiology, VKV American Hospital, Istanbul, TUR; 2 Anesthesiology, Koç University, İstanbul, TUR; 3 Orthopaedics and Traumatology, VKV American Hospital, Istanbul, TUR

**Keywords:** interscalene brachial plexus block, cerebral blood flow, beach-chair position, ultrasound guided regional anesthesia, shoulder arthroscopy, cerebral protective effect

## Abstract

Introduction

Arthroscopic shoulder surgeries are usually performed in a sitting position. The sitting position is known to cause physiological changes related to cardiovascular adaptation. Interscalene nerve blocks (ISB) are the most commonly used techniques and are considered gold standard regional anesthesia methods for shoulder surgeries. Cerebral vessels located around sympathetic ganglia provide sympathetic system integrity. This local anesthetic spreading during ISB could be a side effect or provide a protective effect on cerebral ischemia. Our study aimed to investigate the cerebral protective effect of the ISB in arthroscopic shoulder surgeries in a sitting position.

Material and methods

After the approval of Koç University Clinical Research Ethics Committee (2020.020.IRB1.011), records of patients between January and December 2019 with shoulder arthroscopy at the Vehbi Koç Foundation (VKV) American hospital were retrospectively reviewed. Records of the hemodynamic response, INVOS^TM^ (Medtronic, Minneapolis, USA) (rSO2) parameters, pain scores, and additional analgesic needs of all cases were examined in the intraoperative and postoperative period.

Results

Data of 40 patients who met the criteria to be included in the study was analyzed. Our study showed that the sitting position leading to hypotension coincided with a decrease in INVOS values. Nevertheless, we did not record any significant hypotension after ISB, and this may be due to the use of a minimal dose of local anesthetic. There was a certain increase in near-infrared spectroscopy (NIRS) values ​​after ISB. We saw that the value of regional oxygen saturation (rSO2) increased on both the ISB side and the non-ISB side. This shows that the ISB can have a global impact on the brain. Specificially, the increase in rSO2 values ​​in the ISB side compared to the other side suggests that ISB has possible positive effects on cerebral blood flow.

Conclusion

Our study has shown that ISB may transiently increase the rSO2 levels in the sitting position during shoulder surgery.

## Introduction

Significant acute postoperative pain is common in adults after shoulder surgery, with approximately 45% reporting severe pain in the intermediate postoperative period [1]. Shoulder pain is one of the most common musculoskeletal diseases in adults [2]. Shoulder arthroscopy is a minimally invasive technique applied in various shoulder pathologies and may cause serious pain complaints in the postoperative period. Since the interscalene block (ISB) was defined by Winnie [3] in 1970, it has been used frequently for anesthesia and/or postoperative analgesia, especially in arthroscopic shoulder surgeries. Regional anesthesia techniques have gained importance in terms of anesthesia management to reduce opioid use in shoulder surgeries. Interscalene nerve blocks are the most commonly used techniques and are considered gold standard regional anesthesia methods for shoulder surgeries. ISB may cause Horner’s syndrome resulting from the diffusion of local anesthetic (LA) to cervical sympathetic ganglia. Cerebral vessels are known to be innervated by fibers originating from this sympathetic ganglion [4]. Sympathetic nerve activity regulates cerebral autoregulation. More importantly, an increase in cerebral perfusion may be potentially neuroprotective for silent cerebral ischemia [4]. Arthroscopic shoulder surgeries are usually performed in a sitting position. Many studies report rare but significant neurological injuries such as stroke, spinal cord ischemia, and temporary vision loss in the beach chair position [5]. The incidence of neurologic complications in the beach chair position remains unknown. However, some studies have shown that 0.0004% of such patients have a major stroke [5]. These neurologic complications were reported after shoulder surgery in the beach chair position (BCP) due to hypotension and head above the heart level, perhaps due to cerebral autoregulation failure and cerebral ischemia as a result of decreased cerebral perfusion. The sitting position is known to cause physiological changes related to cardiovascular adaptation. Hypotension occurring after sitting position may cause a decrease in brain blood flow and hypoperfusion [6,7]. Due to the spread of local anesthesia during ISB, it may affect the sympathetic system due to stellate ganglion and cervical plexus involvement [4,7].

In our clinic, shoulder arthroscopies are performed in a sitting or lateral position under general anesthesia combined with regional anesthesia techniques. We use ISB or anterior suprascapular block as a regional anesthesia method. Our study aimed to investigate the cerebral protective effect of the Interscalene brachial plexus block (ISB) in arthroscopic shoulder surgeries in a sitting position.

## Materials and methods

After the approval of the Koç University Clinical Research Ethics committee (2020.020.IRB1.011), records of patients between January and December 2019 with shoulder arthroscopy at the VKV American hospital were retrospectively reviewed. Patients with a history of cerebral events, a history of Alzheimer's-dementia, planned for carotid surgery, and shoulder surgery under emergency or semi-emergency conditions were excluded from the study. In addition, patients who were found to have applied any regional anesthesia method other than ISB were not included in the study. This study was conducted in accordance with the Helsinki Declaration-2013.

The demographic data from the preoperative evaluation forms of the patients and the operation done from the surgical reports were recorded. By reviewing intraoperative anesthesia records, the opioid type and quantities used during the operation were recorded. Records of cerebral blood flow values ​​of cerebral oximetry (INVOS 5100 Cerebral Oximeter, Medtronic, Minneapolis, USA) monitoring were used. Intraoperative anesthesia follow-up forms were examined, and systolic and diastolic pressures and pulse values during the operation ​​were recorded. After the operation, the follow-up forms in the recovery room were examined, and the patients' pain scores (Numerical Rating Scale, NRS) and additional opioid needs were recorded.

Perioperative Anesthesia Management

In shoulder surgery operations performed in a sitting position, the depth of anesthesia (Bispectral Index BIS, Covidien - Medtronic, Minneapolis, USA) and cerebral oximetry (INVOS) parameters are routinely used and recorded at 10-minute intervals. General anesthesia induction and maintenance of the patients are performed under the same protocol with the BIS and INVOS parameters. In all patients, invasive arterial pressure was monitored by inserting a catheter into the radial artery of the contralateral upper extremity before ISB. If remifentanil infusion is needed (changes in hemodynamic parameters secondary to pain, e.g., 10% or more increase in systolic and diastolic pressure, 10% or more increase in heart rate), it is adjusted to a range of 0-250 µg/kg/min. Anesthesia procedures are started in the operating room after all patients have received their informed consent 24 hours before surgery. Two experienced anesthesiologists with more block experience determine the regional anesthesia technique to be applied to patients. ISB is routinely applied in a sitting position before general anesthesia and catheter through the needle method (Contiplex C catheter - B.Braun, Melsungen, Germany) is used for ISB in our clinic. Cervical 5 and 6 (C5-6) nerve roots are displayed with the 15-7 Mhz hockey stick linear ultrasound probe (Siemens CX50, Erlangen, Germany) under ultrasound guidance in the ISB 4 mL of 0.5% bupivacaine (Marcaine, AstraZeneca, USA) is applied around the Cervical 5 nerve root. A catheter is placed in the same area, and postoperative analgesia is provided by a patient-controlled analgesia machine (PCA) by the nerve catheter (0.2% Bupivacaine, infusion rate:5 ml/h, bolus rate:5ml). If any opioid use is required for the maintenance of anesthesia, it is recorded. In our clinic, intraoperative hypotension in shoulder surgeries is planned to be treated with ephedrine hydrochloride (5 mg and as needed) when the mean arterial pressure is below 50 mmHg. All patients are observed in the recovery room for 60 minutes after extubation, and after the pain scores are recorded, they are transferred to their services.

Statistical Methods

Statistical analysis was performed using IBM Corp. Released 2017. IBM SPSS Statistics for Windows, Version 25.0. Armonk, NY: IBM Corp. Shapiro-Wilk’s test investigated the normality of continuous variables. Descriptive statistics were presented using mean and standard deviation for normally distributed variables and median (and minimum-maximum) for the non-normally distributed variables. Non-parametric statistical methods were used for values with skewed distribution. For comparison of two dependent non-normally distributed groups Wilcoxon Signed Rank test was used. Friedman test was used for comparison of more than two dependent non-normally distributed groups. For Post Hoc pairwise comparisons of two dependent nonnormally distributed groups, the Wilcoxon Signed-Rank test with Bonferroni correction was used. Statistical signiﬁcance was accepted when the two-sided p-value was lower than 0.05.

## Results

**Figure 1:**
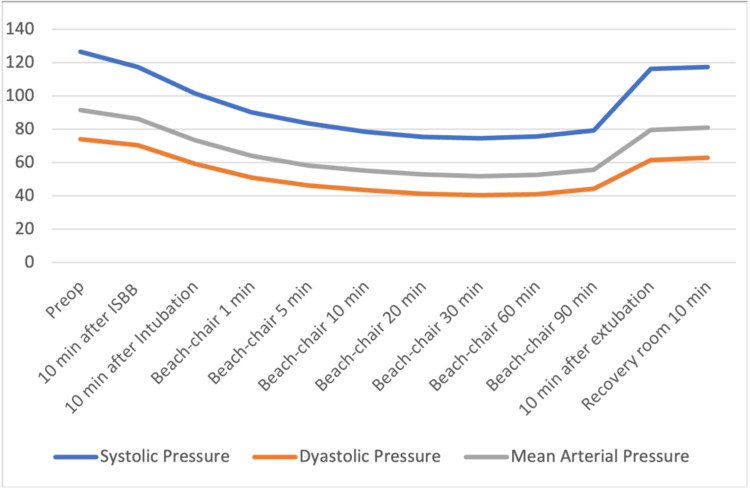
Hemodynamic Responses All times systolic, diastolic and mean values p <0.01 (compared to preop value) Hypotensive effects of the sitting position become particularly evident after 10 minutes after positioning is completed. Stable hemodynamic parameters were observed in the intraoperative period.

**Figure 2:**
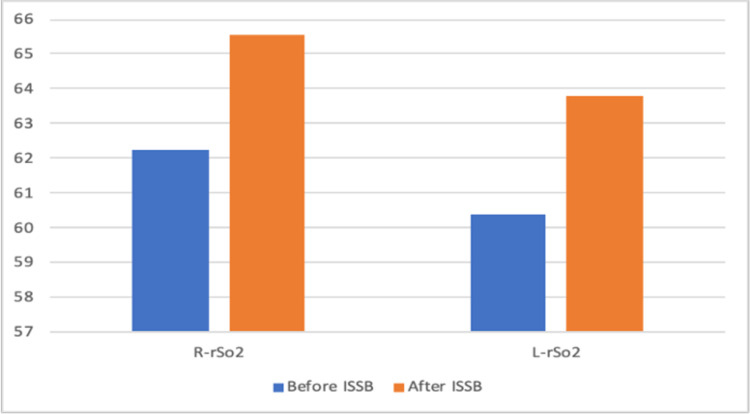
Regional oxygen saturation (rSO2) before and after ISB After ISBB R-rSatO2 p=0.013, After ISBB L-rSatO2 P<0.01 Increase in rSatO2 values ​​after ISBB

**Figure 3:**
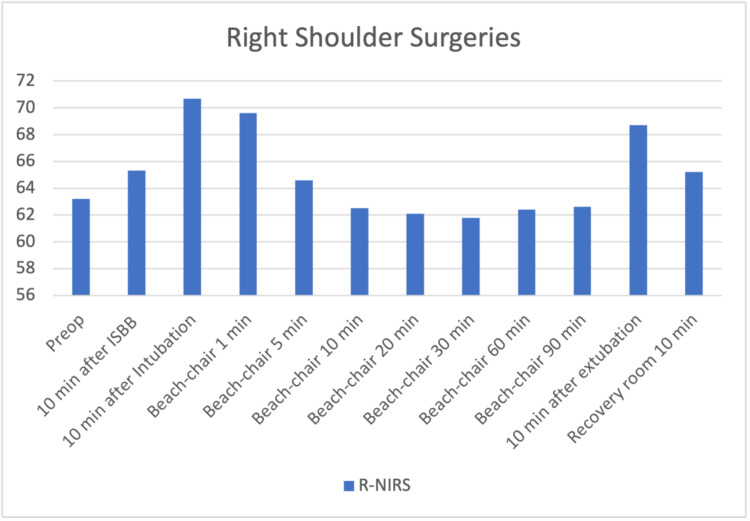
Right shoulder surgeries – right regional oxygen saturation changes 10 min after ISB and Recovery room 10 min increase in rSO2 p<0.05 10 min after Intubation, Beach-Chair 1 min and 10 min after Extubation values p<0.01 No statistical significance between Beach-chair 10 – 90 min values (p>0.05) The increase in rSO2 after ISB ensures that the decrease caused by the sitting position remains close to basal values.

**Figure 4:**
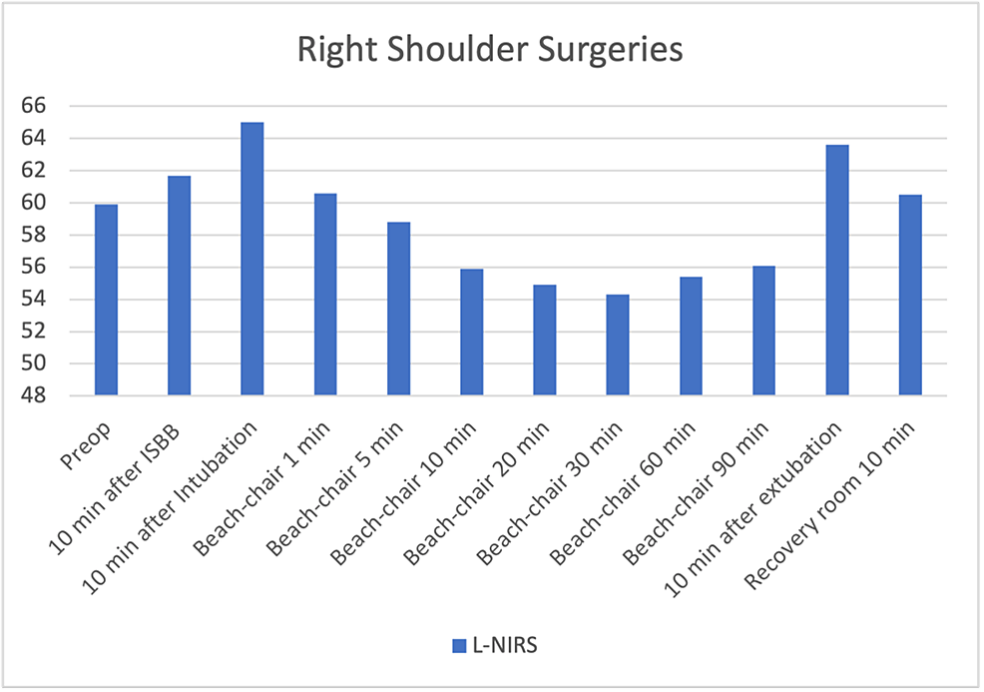
Right shoulder surgeries – left regional oxygen saturation changes 10 min after Intubation and 10 min after Extubation increase in rSO2 p<0.01 Beach-chair 10 – 90 min decrease in rSO2 p<0.01 (compared to preop value) No statistical significance between Beach-chair 10 – 90 min values (p>0.05) The increase in rSO2 after ISB ensures that the decrease caused by the sitting position remains close to basal values. (Even less than the same side)

**Figure 5:**
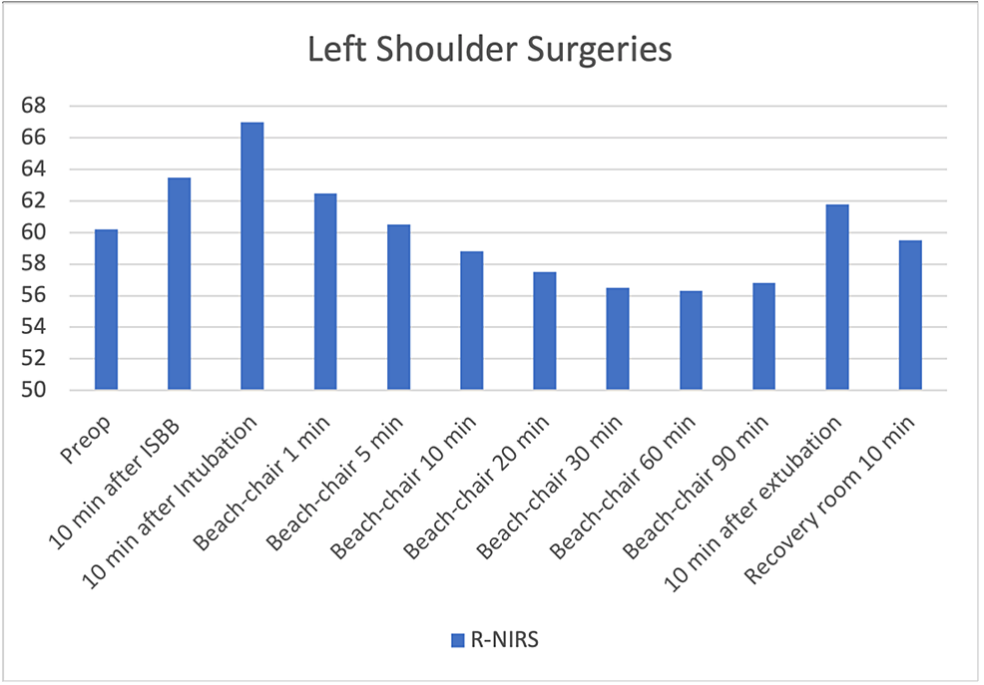
Left shoulder surgeries – right regional oxygen saturation changes 10 min after ISB increase in rSO2 p=0.001 10 min after Intubation increase in rSO2 p=0.001 Beach-chair 1 min increase in rSO2 p=0.003 Beach-chair 10 min decrease in rSO2 p=0.007 Beach-chair 20 min decrease in rSO2 p=0.002 Beach-chair 30 min decrease in rSO2 p=0.002 Beach-chair 60 min decrease in rSO2 p=0.001 Beach-chair 90 min decrease in rSO2 p=0.003 The increase in rSO2 after ISB ensures that the decrease caused by the sitting position remains close to basal values. (Even less than the same side)

**Figure 6:**
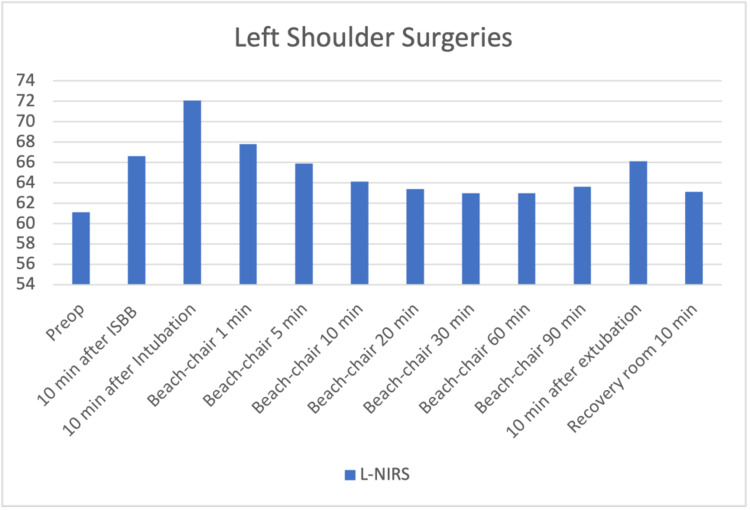
Left shoulder surgeries – left regional oxygen saturation changes 10 min after ISB increase in rSO2 p=0.001 10 min after Intubation increase in rSO2 p=0.001 Beach-chair 1 min increase in rSO2 p=0.001 Beach-chair 5 min increase in rSO2 p=0,001 Beach-chair 10 min increase in rSO2 p=0.006 Beach-chair 90 min increase in rSO2 p=0.045 10 min after Extubation increase in rSO2 p=0.002 The increase on the left side in rSO2 after ISB kept above the basal value after the decrease caused by beach chair position.

The files of 85 patients who underwent arthroscopic shoulder surgery in our clinic between January-December 2019 were retrospectively reviewed. Fifteen patients who were not operated on with the sitting position were excluded. Three patients who had a previous cerebral event and identified as carotid surgery were excluded. One patient with a ventricular-peritoneal shunt was also excluded. Sixteen patients whose INVOS and pain follow-up records were missing were also excluded from the study. The data of 40 patients who met the criteria to be included in the study were analyzed. The patients' demographic graphics and operation features are displayed in Table 1. Hemodynamic changes before and after the operation are displayed in Figure 1. Figure 1 shows a stable hemodynamic balance during surgery. It was observed that there was no secondary response to pain in the 1st and 10th minutes after the surgical incision, such as the absence of sudden increases in systolic and diastolic pressure. This gives information about the effectiveness of the applied regional anesthesia technique. Hypotensive effects of the sitting position become particularly evident after 10 minutes after the sitting position is commenced. When the mean arterial pressures were examined, only 2 patients had values below the critical value of 50 mmHg; it was observed that INVOS values ​​decreased below 40%, which was accepted as the threshold value. In terms of average INVOS values, pre-sedation and post-ISB values ​​increased significantly on the left (Figure 2). Since the dominant hand questioning was not performed in the preoperative evaluation features of the patients, it could not be clarified that this hemisphere could be related to the dominance of the hemisphere of the brain. When individual INVOS values ​​were examined with side surgeries, it was observed that regional oxygen saturation (rSO2) values ​​increased in the same side procedures. However, an increase in rSO2 on the opposite side of the procedure may highlight the possibility of ISB's effects on the global perfusion of the brain (Figure 3-6). It was noted that all patients were given intravenous 1 g paracetamol (Parol, Abdi Ibrahim, Turkey) before extubation. Total additional opioid doses in the intraoperative period and recovery room are shown in Tables 2-3. Postoperative 30 and 60 minutes pain assessments recorded in the recovery room after extubation are shown in Table 4. None of the patients had respiratory distress that could be detected clinically postoperatively.

**Table 1:** Demographic data and operation features (Gender M: Male, F: Female, DM: Diabetes Mellitus, HT: Hypertension and operation side, R: Right, L: Left as the number of patients, other parameters: as the average score) (Age, Height, Weight and Operation time values: mean±standard deviation)

Gender (M/F)	23/17
Age (years)	54.3±9.6
Height (cm)	163.7±6.36
Weight (kg)	73.5±12.21
DM (+/-)	5/35
HT (+/-)	15/25
Operation time (min)	88.7±17.12
Operation side (R/L)	26/14

 

**Table 2:** Perioperative total additional opioid analgesic doses (mg – mean values)

	Tramadol	Morphine	Meperidine
Total opioid amount (mg)	10.5	O	0

**Table 3:** Recovery room total additional opioid analgesic doses (mean values)

	Tramadol (mg)	Fentanyl (mcg)	Meperidine (mg)
Total opioid amount	0	12	10

**Table 4:** Numerical rating scale values for pain Values ​​are given as the number of patients (Number of patients with NRS values ​​between 0-3, 4-6 and 6-10)

	NRS (0 – 3)	NRS (4 – 6)	NRS (6 – 10)
Preoperative	36	3	1
Recovery 30min	33	7	0
Recovery 60min	40	0	0

## Discussion

The sitting position is frequently preferred in upper extremity surgeries, especially arthroscopic shoulder surgeries. Despite the surgical benefits of the sitting position, adverse effects on the patient's physiological variables are well known. Especially, it is accepted that the sitting position may affect cerebral perfusion negatively due to the hydrostatic pressure imbalance between the heart and the brain [6,7]. The physiological point of view demonstrates that changes in cerebral perfusion pressure are most affected by hemodynamic changes in the transition from the supine position to the sitting position. Baroreceptors ensure that the sympathetic system is active and the parasympathetic system inactive when switching from the supine position to the sitting position. The main purpose here is known to be the effort of the body to regulate blood pressure to provide perfusion [8]. In patients under anesthesia, this autonomic response is affected, cerebral perfusion pressure decreases and potentially causes cerebral ischemia due to intraoperative cerebral desaturation. Neurological damages have been reported after sitting position shoulder surgeries with no previous history of cerebral pathology [7,8]. Studies show that even a ten-degree difference in head height creates a difference in blood flow and perfusion pressure [8,9]. When the head position is in the supine position, the middle cerebral artery (MCA) increases the blood flow rate [10]. It is known that changes in position-related cerebral blood flow do not cause a change in regional cerebral oxygen saturation, and brain activity may increase with vascular dilation to increased oxygen demand. Near-infrared spectroscopy (NIRS) measures oxygen saturation using a 75% venous 25% arterial-derived arterio-venous volume ratio. While changes in the arterial and venous volumes of the brain in the supine position may not be very different, venous and arterial volume changes occurring in positions of the head different from the heart level may create differences in the relationship between brain blood flow and NIRS [11,12]. As the transition from lying to sitting position, the sympathetic system is activated due to physiological adaptation in blood pressure, while inactivation occurs in the parasympathetic system. Under anesthesia, the autonomic nervous system response may not be sufficient and cardiovascular adaptation may be impaired [13,14]. Studies on this subject have shown severe hypotension during shoulder surgery can cause permanent and subsequent changes due to cerebral desaturation [15-18]. Our study showed that the sitting position leading to hypotension coincided with a decrease in INVOS values. Considering the negative effects of hypotension on cerebral perfusion, our study showed that sitting position could impair cerebral circulation.

In arthroscopic shoulder surgeries, the sitting position is important for a clearer view of the surgical field. It is important to keep in mind that hypotension is also important to achieve this goal. Hypotension improves the quality of arthroscopic shoulder surgery and also contributes to postoperative recovery. Despite the surgical benefits of hypotension, it should be kept in mind that the patient may be exposed to neurological complications [19]. Non-invasive blood pressure monitoring in sitting position cases may cause errors with an inaccurate estimation of blood pressure values ​​and subsequent interpretation of true cerebral perfusion pressures. Therefore, invasive arterial blood pressure monitoring is recommended for surgery in the sitting position. Neurological complications can range from transient-mild deficits to severe cerebral ischemic damage. Temporary delirium can develop in many cases [17]. For this reason, close and careful follow-up is important in patients at increased risk and sitting positions [16-19]. Keeping the mean arterial pressure between 50 and 65 mmHg also positively correlates to the duration of arthroscopic surgeries [20,21]. During controlled hypotension, brain perfusion, ocular perfusion pressure, and circulation of various organs gain importance. It is thought that circulation in the end-organs can be preserved when the mean arterial pressure is reduced by 30% from the baseline value, with a limit of 50 mmHg [22]. Choi et al. [23] showed that ISB has a positive contribution to hypotension and the clarity of the image in arthroscopic shoulder surgery. The complication rate has decreased with the reduction of the local anesthetic volume in ISB. Probably, using less local anesthetic volume will cause less spread to the cervical sympathetic chain. This may have reduced the possible hypotensive effect. In our study, we did not observe any significant hypotension after ISB, and this may be due to the use of a minimal dose of local anesthetic. In our study, invasive arterial monitoring was used. Therefore, hemodynamic changes during surgery could be monitored effectively.

Aguirre et al. [19] conducted a study on patients undergoing general anesthesia under ASA I - II in a sitting position and demonstrated the frequency of cerebral desaturation events as 25%. They reported that neurological deficits were not observed in patients, but there were negative changes in the neurocognitive evaluation tests. In a large-scale study by Friedmann et al. [17], shoulder position with the sitting position and cerebrovascular accident were primarily related. Cox et al. [24] reported that cerebral desaturation events could not be prevented using INVOS in the shoulder surgeries performed in a sitting position and that intraoperative blood pressure values ​​and INVOS values ​​were connected. In their study, Murph et al. [15] showed that the low values of NIRS were correlated with intraoperative hypotension in sitting position shoulder surgeries, and there was a significant increase in the incidence of cerebral desaturation in these patients. They stated that even if cerebral desaturation develops, NIRS values ​​may be normal, and cerebral blood flow can be better preserved with End-tidal CO_2_ (ETCO2) values ​​between 40 and 42 mmHg in the ventilated patients. Yadeau et al. [25] stated that the regional anesthesia technique reduced the risk of cerebral desaturation events in their study with 99 shoulder surgery operations in a sitting position. Koh et al. [26] found that the frequency of cerebral desaturation events was statistically significantly higher in the group who received general anesthesia than undergoing sedation with interscalene in their study with a 60 sitting position shoulder surgery patient. In many studies, it has been reported that the reason for the lower incidence of cerebral desaturation is the positive effect of moderate hypercapnia caused by sedation in operations performed under regional anesthesia. In our clinic, all shoulder surgeries are performed with a combination of general and regional anesthesia. In the files we investigated, we paid attention to the fact that the ventilation strategies applied to the patients were similar. 

According to our study results, there was a certain increase in NIRS values ​​after ISB. Specifically, the increase in rSO2 values ​​in the ISB side compared to the other side suggests that ISB has significant positive effects on cerebral blood flow. When the data was scanned, no significant decrease was observed in the rSO2 values, even in the mean arterial pressure's 50-60 mmHg values. It shows that the possible increase in rSO2 values ​​after ISB can prevent the rSO2 from reaching the critical limit in hypotension conditions, and the mean arterial pressure corresponding to the critical limit for rSO2 can be interpreted as 50 mmHg. According to the results of our study, after ISB, rSO2 values increase on both the right and left sides. Despite the expected decrease in cerebral blood flow in the sitting position, it may be important to increase these values. Therefore, our results may be interpreted that the ISB preserves cerebral perfusion to a certain extent during hypotension caused by the sitting position in shoulder surgeries. The cervical sympathetic trunk, comprising superior, middle, and inferior ganglia, lies on the prevertebral fascia behind the carotid sheath, close to the spinal roots of the brachial plexus. A lot of studies have indicated stellate ganglion block to be associated with increased CBF [27]. Animal studies demonstrate baroreceptor sympathetic nervous activity (SNA) increases with hypertensive surges during REM sleep, with reflex cerebral vasoconstriction postulated to protect the brain. Therefore, the autonomic tone plays a central role in integrating systemic and cerebral vascular responses [28]. Whether clinical conditions that alter cerebral venous content contribute to ScO2 inaccuracy is uncertain. Further, vasopressors used to defend cerebral perfusion pressure also appear to decrease ScO2 [29]. In our study, no vasopressor was used in any patient.

This study shows that even a small volume of local anesthetic can spread to the cervical plexus or stellate ganglion. As a result, sympathetic block effects may have been observed. A possible explanation of our findings could relate to our targeted injection technique. By targeting the C5 and C6 nerve root of the ISB used in our study, it is possible that LA spread was directed towards the stellate ganglion adjacent to the longus colli. 

Limitations

Our study is a retrospective study. A test for neurocognitive evaluation was not performed in the post-operative period. Although we observed no clinical neurocognitive deterioration, the available data cannot objectively support this. A monitoring method was not used to determine whether all patients had sufficient intravascular volume. Although the fasting period of all patients is the same (clinical protocol) and the fluid management during the operation is the same type, this is not scientifically clear. The effect of fluid resuscitation on brain perfusion may be one of the missing points in the study since the vessel volumes of the patients were not evaluated. Lung protective ventilation strategies are used in our clinic. However, the same ventilation modes were not used in all patients in the study as this was not a prospective study.

## Conclusions

Our study has shown that the ISB can increase the rSO2 levels in the brain in sitting position shoulder surgery. This increase in rSO2 values can be interpreted as increased cerebral perfusion. Specifically, increased rSO2 on the non-ISB side may be important in terms of global perfusion. Perfusion increase may prevent signs of cerebral desaturation. In conclusion, performing preoperative ISB is an important criterion in shoulder surgeries to decrease the incidence of cerebral desaturation.
